# Characterization of Morphological and Cellular Events Underlying Oral Regeneration in the Sea Anemone, *Nematostella vectensis*

**DOI:** 10.3390/ijms161226100

**Published:** 2015-12-01

**Authors:** Aldine R. Amiel, Hereroa T. Johnston, Karine Nedoncelle, Jacob F. Warner, Solène Ferreira, Eric Röttinger

**Affiliations:** Institute for Research on Cancer and Aging, Université de Nice Sophia-Antipolis UMR 7284, INSERM U1081, CNRS UMR 7284, Nice 06107 Cedex 02, France; aldine.amiel@unice.fr (A.R.A.); j.hereroa@hotmail.fr (H.T.J.); Karine.Nedoncelle@unice.fr (K.N.); warner.jacob@gmail.com (J.F.W.); solene.ferreira.ca@gmail.com (S.F.)

**Keywords:** regeneration, wound healing, tissue tracking, marine invertebrate, sea anemone, *Nematostella vectensis*

## Abstract

Cnidarians, the extant sister group to bilateria, are well known for their impressive regenerative capacity. The sea anemone *Nematostella vectensis* is a well-established system for the study of development and evolution that is receiving increased attention for its regenerative capacity. *Nematostella* is able to regrow missing body parts within five to six days after its bisection, yet studies describing the morphological, cellular, and molecular events underlying this process are sparse and very heterogeneous in their experimental approaches. In this study, we lay down the basic framework to study oral regeneration in *Nematostella* *vectensis.* Using various imaging and staining techniques we characterize in detail the morphological, cellular, and global molecular events that define specific landmarks of this process. Furthermore, we describe *in vivo* assays to evaluate wound healing success and the initiation of pharynx reformation. Using our described landmarks for regeneration and *in vivo* assays, we analyze the effects of perturbing either transcription or cellular proliferation on the regenerative process. Interestingly, neither one of these experimental perturbations has major effects on wound closure, although they slightly delay or partially block it. We further show that while the inhibition of transcription blocks regeneration in a very early step, inhibiting cellular proliferation only affects later events such as pharynx reformation and tentacle elongation.

## 1. Introduction

Regeneration is the biological process that enables organisms to regrow missing body parts after amputation. This fascinating phenomenon has intrigued naturalists and scientists for more than 300 years. Among the first animals in which regeneration has been described was the freshwater polyp *Hydra*, a cnidarian that belongs to the group of hydrozoans. While regenerative potential has since then been described in other groups of cnidarians (Anthozoa [1,2], Cubozoa [3], and Scyphozoa [4]), the majority and most detailed cellular and molecular regeneration studies in this phylum have been carried out using Hydrozoa (reviewed in [5,6]). These studies highlight variations in the cellular mechanisms (e.g., cellular proliferation) underlying regeneration in different groups of cnidarians (the hydrozoan *Hydra* *vs.* the anthozoan *Nematostella* [7]). They also demonstrate variations within Hydrozoa (*Hydra* *vs.* *Hydractinia* [8]) and even within the same species; in *Hydra*, for example, the regenerative mechanism varies depending on the amputation site [9]. Therefore, additional systems are required to determine whether common mechanisms govern regeneration throughout Cnidaria and to understand the evolution of this process within the animal kingdom.

**Figure 1:**
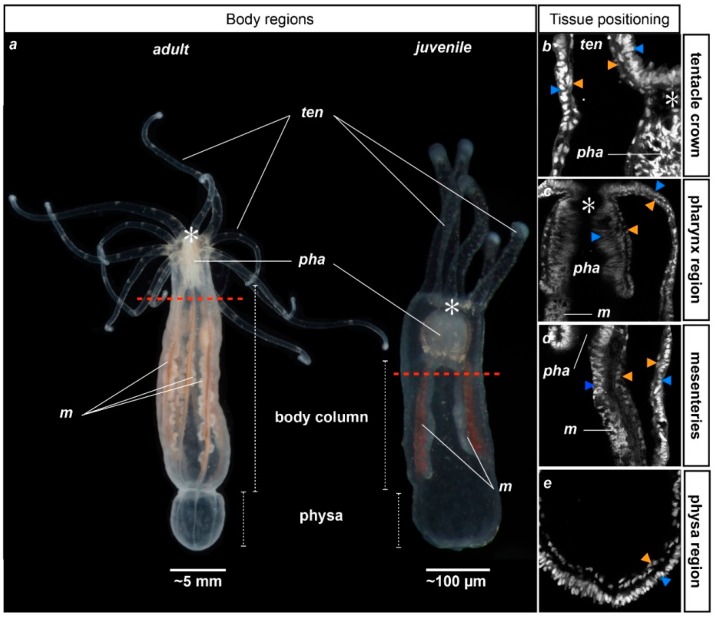
General anatomy of adult and juvenile *Nematostella vectensis*. Photographs illustrating the adult (**left**) and juvenile (**right**) morphology of *Nematostella*; (**a**) Polyps are oriented toward the oral region to the top and aboral region to the bottom. Red dotted lines indicate the future amputation site for the following experiments of this study. (**b**–**e**) Close-up confocal stack images of the tentacle (**b**); pharyngeal (**c**); mesenterial (along the body column) (**d**); or the physal (**e**) regions. Juvenile polyps were stained with DAPI to label the nuclei (white staining). An asterisk (*) indicates the position of the mouth. Orange and blue arrowheads indicate the gastrodermal and ectodermal epithelia, respectively. *ten*, tentacles; *pha*, pharynx; *m*, mesenteries.

The majority of cnidarian species belong to the Anthozoa class (sea anemones and corals) which diverges basally to their other cnidarian sister groups [10,11,12]. This places anthozoans at a key phylogenetic position to understand the evolution of the regeneration process among Cnidaria. Several anthozoans have been shown to display high regenerative capacities [1,2,13]. However, very little is known about the cellular/molecular mechanisms that underlie this process in these animals. The anthozoan sea anemone *Nematostella vectensis* (*Nv*) is a well-established system for the study of embryonic and larval development [13,14,15,16,17,18]. Interestingly, it is also able to regrow half of its oral or aboral body within five to seven days after bisection (Figure 1) [12,13].

*Nematostella* is a diploblastic animal comprised of an outer ectodermal epithelium and an inner gastrodermis that are separated by muscle fibers and the mesoglea (Supplementary Figure S1A,B). The oral region consists of a pharynx and tentacles that surround the mouth opening. Freshly metamorphosed juveniles possess four tentacles that are used to catch food that floats by, while adult polyps have up to 16 [19]. The body column (or scapus) ends in its aboral-most region in a structure that is called the physa (Figure 1; [20,21,22]). Inside the body cavity, internal structures termed mesenteries stretch from the pharynx to the physa and correspond to the digestive and reproductive organs of the animal (Figure 1). Two primary mesenteries are distinctly visible in juveniles, while eight mesenteries that produce the germ cells are found in adult *Nv *(Figure 1) [23].

Existing resources [24,25,26,27] as well as cellular and molecular tools developed by the *Nematostella *community (reviewed in [28], Layden *et al.*, submitted) have over the past few years increased the interest to study regeneration in this cnidarian. However, studies describing the morphological, cellular, and molecular events underlying this process are still sparse and very heterogeneous in their experimental approaches [7,12,13,29,30,31,32,33,34,35]. In particular, different studies used (i) animals of different ages (juveniles *vs.* adults); (ii) different amputation sites (sub-pharyngeal, supra-physa); and (iii) isolated body parts were left to regenerate at different temperatures (between 15 and 27 °C). The potential to regenerate into a functional organism appears to be similar between the different body parts of an adult *Nv* [12]. The temperature aspect has recently been addressed by Bossert and colleagues, who showed in a study describing a *Nematostella *staging system for the regenerating adult isolated physa (NRSS) that the regeneration speed is temperature-dependent [30]. However, no particular reason was mentioned for the choices of the above-mentioned criteria by the authors; importantly, there is nothing currently known about the influence of age on the regenerative capacity and/or cellular mechanism deployed by *Nematostella *to reform the missing body parts.

The majority of the studies on *Nematostella* regeneration have focused on oral regeneration following sub-pharyngeal amputation. In our present study, we show that there are no apparent differences in the cellular mechanisms underlying oral regeneration after sub-pharyngeal regeneration in juveniles compared to adults. We further lay down the basic framework to study oral regeneration in juvenile *Nematostella *by taking advantage of the ease of animal imaging at this stage. We carefully analyze and describe morphological and cellular events that define specific landmarks of regeneration. Additionally, we propose assays to assess wound healing success and pharynx re-formation. Finally, we show the usefulness of these assays and landmarks for phenotype characterization from perturbation experiments.

## 2. Results

### 2.1. Head Regeneration Is Temperature—But Not Age-Dependent

**Figure 2:**
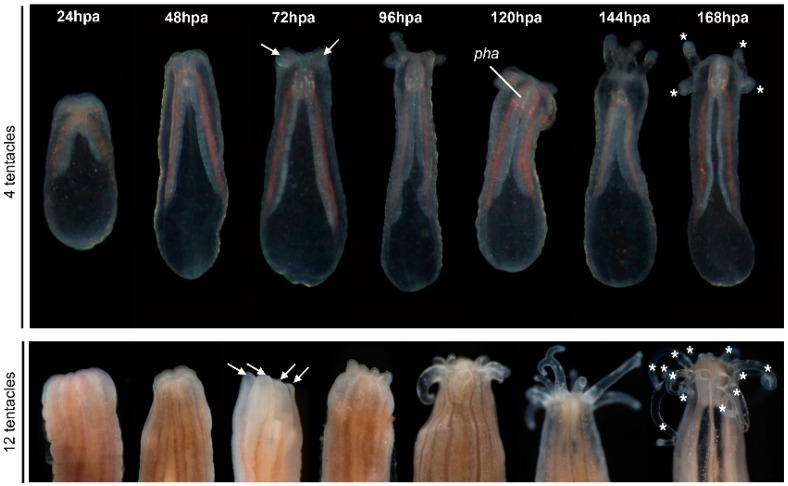
Timing of oral regeneration is similar in juveniles and adults. Comparison of the duration of oral regeneration between six-week-old juveniles (**upper** panel—four tentacles) and adults (**bottom** panel—12 tentacles). From left to right: regenerating polyps at 22 °C 24 h post-amputation (hpa), 48, 72, 96, 120, 144 and 168 hpa. At 72 hpa, in both juvenile and adult polyps, the tentacles bulbs are clearly visible (white arrows), and the pharynx starts to form in some individuals (only visible in transparent juveniles). Five days post-amputation (120 hpa), the juvenile and adult polyps are regenerated as indicated by the presence of the pharynx and elongated tentacles. The white asterisk at 168 hpa indicates the tentacles: four on the regenerating juvenile and 12 on the regenerating adult polyp.

While the age of adult *Nematostella* cannot yet be determined at a molecular or cellular level, one can distinguish juvenile and adult polyps from their anatomy (Figure 1). Previous studies describing regeneration in *Nematostella* have been carried out either in juveniles [7,34] or in adults [12,29,30,31,32,33,35]. However, it is not known if the regenerative capacities/mechanisms are conserved between the two. In order to determine if the timing of oral regeneration in *Nematostella* is age-dependent, we performed head amputation (bisection under the pharyngeal region; see red dotted line in Figure 1a) experiments in *Nematostella* juveniles and adults while following the timing of regeneration. This sub-pharyngeal amputation site was used in all of the following amputation experiments performed in this study. We analyzed the morphology of the regenerating animals at 22 °C (temperature used in [7]) each day for one week. We observed that both juveniles (20 out of 20 cases) as well as adults (10 out of 10 cases) regenerate with a similar timing (Figure 2).

We observed a specific sequence of events during the regeneration process (Figure 2). First, the mesenteries come into tight contact with the amputation site 24–48 h post-amputation (hpa, only observable in transparent juveniles). Next, at 72–96 hpa the tentacle bulbs become visible in juveniles and adults. Finally, there is a progressive elongation and formation of those structures up to 144 hpa (Figure 2). Interestingly, we also observed that the number of new regenerating tentacles corresponds to the number of tentacles present in the polyp before amputation. The four-tentacle juveniles regenerate four new tentacles and the 12+ tentacle adults regenerate 12+ new tentacles (Figure 2, 168 hpa).

In order to investigate if oral regeneration following sub-pharyngeal amputation is temperature-dependent as a function of age, we performed sub-pharyngeal bisections in *Nematostella* juveniles or adults and left them to regenerate at 16 °C (Supplementary Figure S2A,B). The timing of oral regeneration for both juveniles (20 out of 20 cases, Supplementary Figure S2A) and adults (10 out of 10 cases, Figure S2B) is considerably slowed at 16 °C compared to 22 °C. In particular, the appearance of the tentacle bulbs is delayed by approximately 48–72 h in animals that regenerated at 16 °C. This observation is in line with a previous report that showed that the timing of regeneration for the isolated adult physa is temperature-dependent [30]. All following experiments were carried out at 22 °C for consistency and to be able to compare our findings to those reported by Passamaneck and Martindale and Bossert and colleagues [7,30].

### 2.2. Cell Proliferation Is Required during Head Regeneration in Adult Tissue

**Figure 3:**
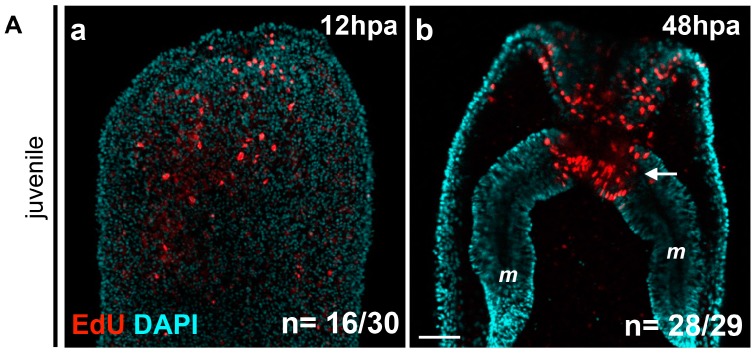

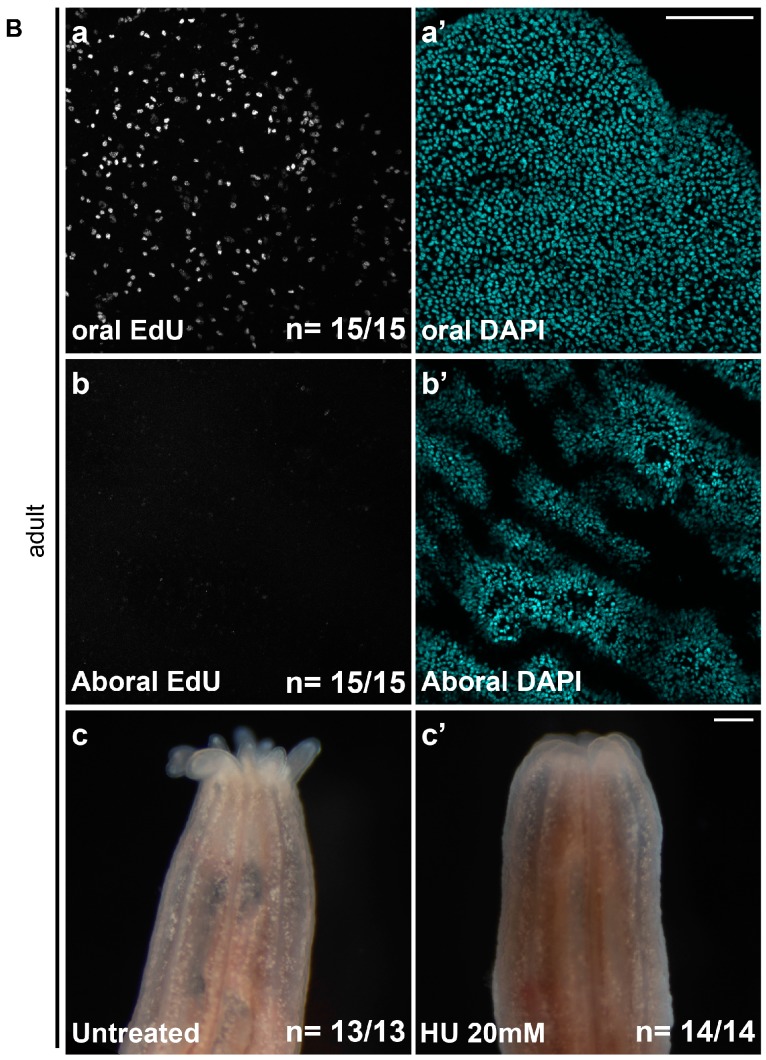
(**A**) Localized cellular proliferation in juveniles begins at 12 hpa at the amputation site. Overlapping confocal images in which the nuclei (DNA, cyan) were stained using DAPI and proliferating cells (red) were marked using an EdU labeling kit (**Aa**,**Ab**). Oral parts of the regenerating juveniles at 12 hpa (**Aa**) and 48 hpa (**Ab**). All animals are oriented with the amputation site to the top. The white arrow in (**b**) shows the presence of dividing cells in the most oral part of the mesentery tissues. Number of cases for the representative phenotype are in white at the bottom right of each image. *m*, mesentery; (**B**) Cell proliferation is necessary for adult tissue regeneration. Cell division is present during regeneration in adults after sub-pharyngeal amputation (**Ba**–**Bb’**). (**Ba**–**Bb’**) Confocal stack images in which the DNA (nucleus) is labeled with DAPI (cyan) and proliferating cells (white) were marked using an EdU labeling kit. (**Bc**,**Bc’**) Blocking cell proliferation using hydroxyurea (HU) blocks regeneration in the adult amputated polyps (**c’**) contrary to the regenerated untreated control (**c**). Number of cases for the representative phenotype are in white at the bottom right of each images (**Ba**,**Bb**,**Bc**,**Bc’**). Scale bar in (**Ab**) is 20 μm and applies to (**Aa**). Scale bar in (**Ba’**) is 50 μm and applies to (**Ba**,**Bb**,**Bb’**). Scale bar in (**Bc’**) is 1 mm and applies to (**Bc**).

In *Nematostella*, cell proliferation is required for head regeneration in juveniles [7]. In order to determine the earliest time-point where this cellular proliferation is detectable, we performed EdU labeling at 1.30, 6, 12, 24, and 48 hpa. We found that while no staining is detectable at the two earliest time-points (data not shown), clear cellular proliferation is observed at 12 hpa at the amputation site (Figure 3Aa).

Cellular proliferation increases massively in this region between 24 hpa and 48 hpa, confirming a previous report (Figure 3Ab) [7]. Interestingly, and in contrast with previous observations, EdU-positive cells were not only detected in the ectodermal epithelium and the gastrodermis at the amputation site, but also in the oral-most regions of the mesenteries (Figure 3Ab).

In order to investigate if cell proliferation is also detectable during adult regeneration [7], we performed EdU staining on adult polyps 48 h after sub-pharyngeal amputation (Figure 3(Ba–Bb’)). While no EdU-positive cells are detected in aboral tissues (Figure 3(Bb,Bb’)), cellular proliferation is clearly visible at the amputation site in the adult tissue at 48 hpa (Figure 3(Ba,Ba’)). We further tested if cellular proliferation, similarly to juveniles [7], is required for adult regeneration. To do this we used the pharmaceutical cellular proliferation inhibitor hydroxyurea (HU). Continuous treatment of HU for six days post-amputation blocks oral regeneration in the adult polyp (Figure 3(Bc,Bc’)). Thus, cell division is also required for oral regeneration in adult *Nematostella.*

### 2.3. Wound Healing Occurs at 6 HPA

**Figure 4:**
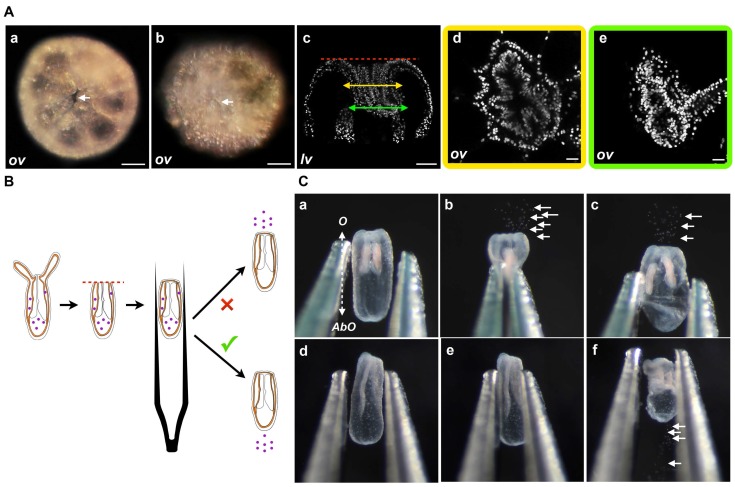
(**A**) Wound closure. Oral opening during *Nematostella* regeneration. Example of DIC images of the oral part, oral view (*ov*), at the amputation site of an example of the opening at 2 hpa (white arrows in (**Aa**)) or the closed wound at 6 hpa (white arrows in (**Ab**)); (**Ac**–**Ae**) are confocal images of the oral-most part of the same polyp in lateral view (*lv*) or details of the oral view (**Ad**,**Ae**); (**Ad**) (yellow frame) or (**Ae**) (green frame) correspond to the double yellow or green arrow slice, respectively, in (**c**); Because of the folding that occurs at the amputation site during the first hours of regeneration, the dynamics of the wound healing are hard to assess in DIC optic or confocal images. Scale bars are 20 μm in (**Aa**–**Ac**) and 10 μm in (**Ad**,**Ae**); (**B**) Diagram of the compression assay during regeneration. The purple dots represent the nematosomes. The red dotted line represents the amputation site. The forceps are laterally compressing the regenerating polyp body; (**C**) Time series of the compression assay in an opened (**Ca**–**Cc**) or a wound-closed (**Cd**–**Cf**) polyp. The dotted double arrow in (**Ca**) indicates the axial orientation of the animals shown in (**Ca**–**Cf**). *O*, Oral; *AbO*, Aboral.

A recent report analyzed *Nematostella* wound healing after puncturing the body column with a glass needle [34]. The authors found that this process is visually completed after approximately 4 h (25 °C). In order to determine exactly when wound healing occurs in *Nematostella* juveniles, we performed DIC and confocal imaging on regenerating juveniles following sub-pharyngeal amputation at several time-points. While in some cases we were able to visualize a clear opening using DIC optics (Figure 4Aa), the three-dimensional folding and contraction of the tissues at the amputation site often made it hard to distinguish between a real wound or a depression that looked like an open wound (Figure 4(Ab–Ae)). Thus, determining the wound closure simply by imaging proves to be difficult. In addition, using classical staining/imaging approaches we are unable to distinguish between a closed wound or contraction of the surrounding myo-epithelia.

In order to have a more robust way to address wound healing after sub-pharyngeal amputation, we developed a compression assay to assess the state of the opening at the amputation site. This assay uses nematosomes (Supplementary Figure S3) as a marker to follow the fluid dynamics present in the gastric cavity of *Nematostella* (Figure 4B,C).

**Figure 5:**
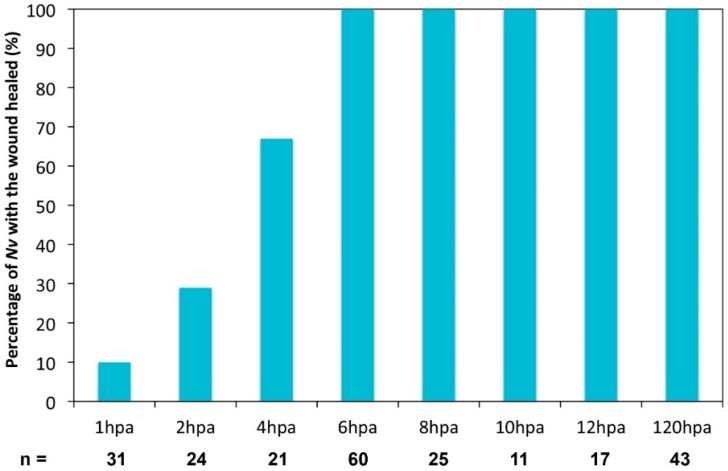
Wound healing assay.

Nematosomes are cellular aggregates formed by cnidocytes that are freely circulating within the *Nematostella* body [36]. When compressing a juvenile with an open wound, the nematosomes will be expelled at the amputation site. On the contrary, when the wound is closed, the nematosomes will either remain in the gastric cavity or leak out of the body cavity through the aboral pore. This pore is an opening with an unknown function located at the aboral-most region of the body (Supplementary Figure S4) [37]. We assume that nematosomes will exit the body cavity through the wound or the aboral pore depending on the wound healing status. To use this wound healing assay, we compressed the body column of sub-pharyngeal amputated juveniles at 0, 1, 2, 4, 6, 12 hpa and followed the behavior of the nematosomes (Figure 5).

Interestingly, at 1 and 2 hpa in the majority of cases (90% *n* = 31 and 71% *n* = 24, respectively), the nematosomes are forced to exit the body cavity through the amputation site (Figure 5), showing that the wound is not healed yet. However, at 4 hpa, the majority of the juveniles (67%, 14 out of 21 cases) exhibited nematosomes leaking through the aboral pore. This is the case for 100% of juveniles (22 out of 22 cases) at 6 hpa and later time-points (Figure 5). Thus, in *Nematostella*, the wound is closed between 4 and 6 hpa following a sub-pharyngeal amputation.

### 2.4. Mesentery Behavior and Pharynx Formation as Specific Landmarks for Oral Regeneration

**Figure 6:**
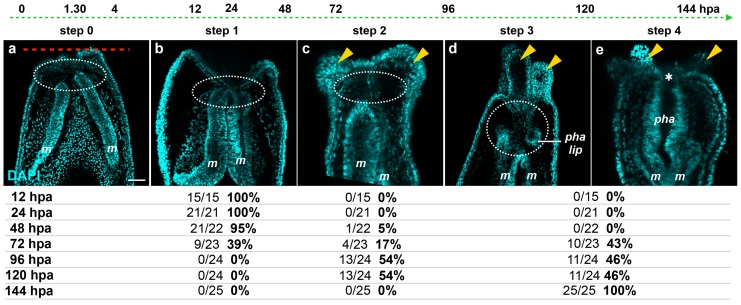
Dynamics of the oral tissue during regeneration. The dynamic behavior of the oral tissue during regeneration was analyzed using confocal microscopy. (**a**–**e**) Confocal image stacks in which the DNA (nucleus) is labeled with DAPI (cyan). Five main phenotypes were observed between 0 and 144 hpa and are represented in this figure: (Step 0) the remaining parts of the mesenteries are separated together and from the epithelia at the amputation site (white dashed circle) (**a**); (Step 1) the remaining parts of the mesenteries are fused together and are in tight contact with the epithelia at the amputation site (white dashed circle) (**b**); (Step 2) an empty space forms between the remaining part of the mesenteries and the amputation site (white dashed circle) (**c**); (Step 3) the pharyngeal lip forms at the oral-most region of the remaining mesenteries, and the empty space becomes filled with nuclei (white dashed circle) (**d**); (Step 4) the pharynx is fully formed at the oral-most region of the remaining mesenteries (**e**). Numbers at the bottom of the image panel indicate the total number of analyzed specimens at 12, 24, 48, 72, 96, 120, 144 hpa and the number of cases representative of one of the described five steps in relation to the regeneration time in hours post-amputation (hpa). The amputation site is represented by a red dashed line in (**a**). Tentacle bulbs and elongated ones are shown by the yellow arrowhead (**c**–**e**). The white asterisk is the mouth opening (**e**). *m*, mesentery; *pha lip*, pharyngeal lip; *pha*, pharynx. Scale bar in (**a**) is 20 μm and applies to (**b**–**e**).

The size and transparency of juvenile *Nematostella* make them more amenable for detailed imaging experiments than adults. We thus analyzed in detail the tissue behavior during oral regeneration of juveniles. We performed DNA labeling on regenerating juveniles following sub-pharyngeal amputation and observed the sequential events every 12 h from 0 to 144 hpa using confocal imaging (Figure 6).

Focusing on the behavior of the mesenteries and the pharynx reformation, we distinguish four main characteristic features: (Step 0) 0–12 hpa, no contact between the remaining mesenteries and the surrounding oral epithelia (Figure 6a); (Step 1) 12–48 hpa, contact of the remaining mesenteries between each other at their most oral site and with the surrounding epithelia at the amputation site (Figure 6b); (Step 2) 60–96 hpa, emergence of a space between the mesenteries and the epithelia at the amputation site (Figure 6c). The epithelia of the amputation site seems dragged down towards the aboral region by the remaining mesenteries. This accentuates the protrusion of the developing tentacle bulbs; (Step 3) 72–120 hpa, the pharyngeal lip (basal part of the future pharynx) forms (Figure 6d). Interestingly, the pharyngeal lip appears to develop first from the oral-most part of the remaining mesenteries; (Step 4) 96–144 hpa, the pharynx is fully regenerated and the tentacles elongate (Figure 6e). Subsequently, the upper part of the pharynx forms (Step 4) progressively in the space that was previously created (Figure 6e) and corresponds to a highly proliferative region (Figure 3Ab).

### 2.5. Fluorescence in the Pharyngeal Region as a Landmark for Pharynx Reformation

**Figure 7:**
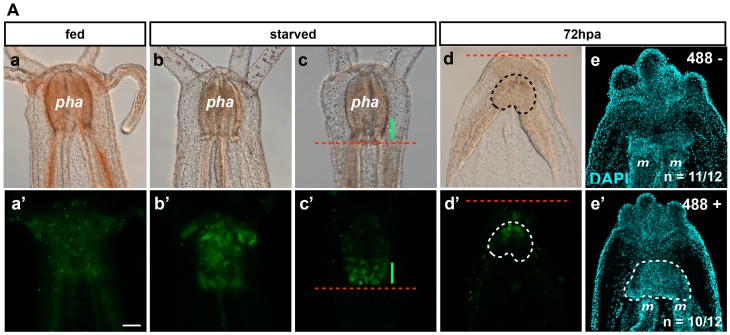

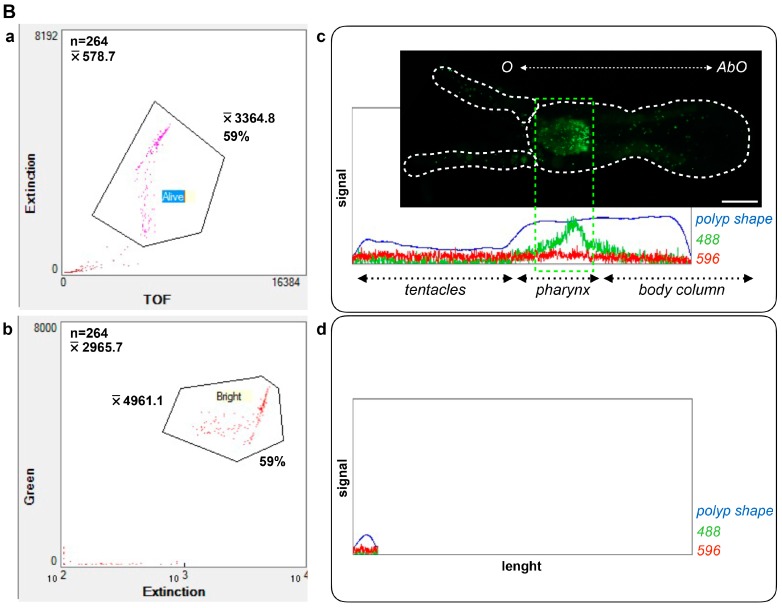
(**A**) Image of 488+ detection in the pharynx. Fed (**a**,**a’**), starved (**b**–**c’**), regenerating 72 hpa (**d**–**e’**) *Nematostella* polyp juveniles. DIC optic images (**a**–**d**). Epifluorescent images (**a’**–**d’**). The red dotted line labels the amputation site under the pharynx (**c**–**d’**). The green line in (**c**,**c’**) shows the 488+ fluorescence localized in the basal part of the pharynx. The area delimitated with the dotted line in **d** and **d’** is the region where the 488+ re-emerged in the polyp at 72 hpa. (**e**,**e’**) Confocal images at 72 hpa labeled for DNA (nuclei in cyan) on the 488-negative (**e**) and 488-positive (**e’**) polyp juvenile. The area delimitated with the white dotted line in (**e’**) (488+ regenerating polyps) shows the pharyngeal lip/pharynx in formation that is absent from the 488-negative regenerating polyps in which only the contact between the two mesenteries is visible. *pha*, pharynx; *m*, mesentery. Scale bar in (**Aa’**) is 20 μm and applies to (**Aa**–**Ae**,**Ab’**–**Ae’**); (**B**) Biosorter. The dot plot in (**a**,**b**) contains the sorting results of the animal by density (Extension) *vs.* mass (Time Of Flight—TOF) or by 488 fluorescence intensity (Green) *vs.* density (Extension), respectively, using a Biosorter system. (**c**,**d**) Examples of the Biosorter profiles (signal *vs.* length) of the bright fluorescent group (Bright in **b**, which corresponds to the Alive group in (**a**)) or the non-fluorescent group (unlabeled group localized at the bottom left of the two dot plots (**a**,**b**)). *O*, Oral; *AbO*, Aboral. Scale bar is 100 μm in (**Bc**).

*Nematostella*, like other cnidarians, possesses endogenous fluorescence emitted by fluorescent proteins and/or fluorescence of the tissues. The six-week-old *Nematostella* juveniles display a green (excitation wave length at 488 nm) fluorescence (henceforth referred to as 488+) from a currently unknown origin (Figure 7A).

The 488+ is randomly distributed throughout the entire body in freshly fed animals (Figure 7(Aa,Aa’)). Interestingly, it became more and more localized to the pharynx when juvenile polyps were starved for one or two weeks (Figure 7(Ab,Ab’,Ac,Ac’)), suggesting a correlation of this staining with a metabolic state of the animals. The varied metabolic states within one batch of animals could explain the asynchrony of regeneration. It may also contribute to differences in the cell proliferation rate observed between animals (see table below Figure 6; Röttinger, unpublished data). After sub-pharyngeal amputation, we observed that the 488+ re-emerges in the regenerating polyp in nearly half of the cases around 72 hpa (Table 1).

**Table 1:** The 488+ in hand-sorted regenerating polyps. Counting of the 488+ polyp juveniles at 24, 48, and 72 hpa. Two different experimenters performed blind counts. *Hand-sorter *1 indicates the first experimenter and *Hand-sorter 2* the second.

–	*488*	24 hpa		48 hpa		72 hpa	
Hand-sorted	+	0/41	0%	4/66	6%	44/77	57%
	−	41/41	100%	62/66	94%	33/77	43%
Bio-sorted	+	3/41	7%	2/66	3%	31/77	40%
	−	38/41	93%	64/66	97%	46/77	60%

We thus used the localized green fluorescence in the pharynx of the uncut animal as a proxy to harmonize a batch of polyps before a series of cutting experiments. Sorting batches of uncut animals was performed using a large particle flow cytometer (Biosorter system, Union Biometrica) that enables the analysis of animals based on their length (time of flight, TOF), density (extinction), morphology (profiler), fluorescence, and the relative localization of the fluorescence along the animal body. In order to analyze the global amount of fluorescence intensity and its localization within the animals, we first defined debris (41% of the population; *n* = 264) based on morphology parameters (TOF and extinction parameters of each polyp; dot plot Figure 7Ba). We then measured the 488+ fluorescence intensity (the mean of fluorescence intensity is *mfi* = 4961.1) within the same batch (59% of the population; *n* = 264; dot plot Figure 7Bb). This 488+ can be localized within the profile of the animal using the Profiler software (Profile Figure 7Bc). Strikingly, the highest amount of 488+ fluorescence is localized in the region of the polyp where the pharynx is supposed to be.

In order to sort them in an automatic manner with the Biosorter, we identified a profile of interest (Figure 7Bc). We then bio-sorted 84 juveniles with this selected profile, performed sub-pharyngeal amputation, and followed their regeneration. In parallel, we hand-sorted 31 animals that size-matched and appeared in good condition without the use of the green auto-fluorescence proxy. All animals were cut below the pharynx, removing the 488+ at 0 hpa. Animals were then placed back into culturing conditions and the re-emergence of the 488+ was assessed in the regenerating polyps from hand-sorted *vs.* bio-sorted batches (Table 2).

**Table 2:** Emergence of the 488+ fluorescence during oral regeneration in the hand-sorted *versus* bio-sorted polyps at 72 and 120 hpa.

–	*488*	72 hpa		120 hpa	
Hand-sorted	+	14/33	42%	18/31	58%
	−	19/33	58%	13/31	42%
Bio-sorted	+	38/84	45%	51/84	61%
	−	46/84	55%	33/84	39%

Interestingly, both batches, hand-sorted *vs.* bio-sorted, displayed a similar heterogeneity in their individual advancement through the regeneration steps as reflected by the numbers of 488+ *vs.* 488− polyps in each batch of animals (Table 2). While the 488+-based selection did not yield a better or more synchronous regeneration, this experiment shows that the Biosorter system can be used as a tool to physically sort animals with precise criteria and/or analyze them for a specific phenotype in large-scale experiments in an unbiased manner. These data also show that the viability of the animals and their regeneration rate are not affected by the Biosorter system.

In order to determine if a correlation exists between the emergence of the 488+ and any previously described steps of regeneration, we sub-pharyngeally bisected juveniles and analyzed the phenotypes in detail of the 488+ and 488− polyps at 72 hpa. We observed that the 488+ starts to emerge in the oral-most part of the remaining mesenteries around 72 hpa (Figure 7(Ad,Ad’)). Using confocal imaging on DAPI-stained (nucleus) animals we analyzed the detailed morphology of 488− and 488+ regenerating juveniles at 72 hpa. We observed that 488− are mainly at Step 2 (11 out of 12 cases) of the oral regeneration staging system described in Figure 6 and Figure 7Ae. No pharynx in formation is visible. Interestingly, the 488+ are mainly at Step 3 or 4 (10 out of 12 cases), with a clear pharyngeal lip or pharynx in formation (Figure 7Ae’). These observations show a strong correlation between the initiation of pharynx formation (Steps 3 and 4) and the presence of 488+ in the regenerating juvenile after sub-pharyngeal amputation.

**Figure 8:**
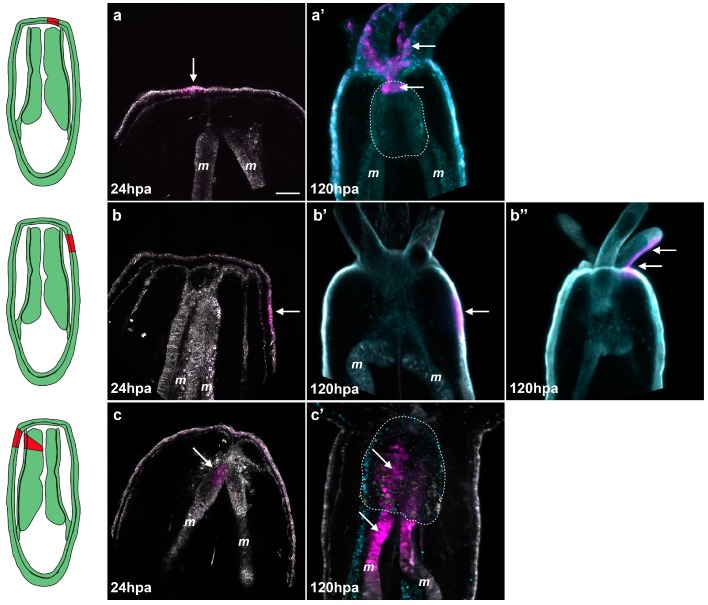
Tissue tracking experiment during regeneration. Spectral confocal images of *Nematostella* juveniles expressing Kaede photoconvertible fluorescent protein mRNA (**a**–**c’**). The Keade photoconverted region is represented in magenta and the non-photoconverted region in grey. Endogenous fluorescence is shown in turquoise to help visualize the morphology of the polyp (**a’**–**c’**); At 24 hpa, regions of interest (the epithelium central to the amputation site (**a**); the epithelium lateral to the amputation site (**b**), or the oral tip of the mesenteries (**c**)) were exposed to a UV laser resulting in permanent photo-conversion of the Kaede protein (magenta); The photoconversion at the central epithelium reveals integration of the converted patch (white arrows in (**a’**)) into the tentacles and most oral tip of the pharynx (white dotted line); Photoconversion of the lateral epithelium shows that this tissue remains in place during regeneration (white arrow in (**b’**)) or is incorporated into the adjacent tentacles (white arrows in (**b’’**)); The photoconversion of the oral tip of the mesentery results in integration of the converted patch (white arrows in (**c’**)) into the pharynx (white dotted line). Scale bar is 20 μm in (**a**).

### 2.6. The Regenerating Pharynx Forms from the Oral Part of the Remaining Mesenteries

Taken together, our observations of the sequential events (Figure 6) and the 488+ emergence (Figure 7A) during regeneration led us to hypothesize that the regenerating pharynx after sub-pharyngeal amputation may come from the oral-most part of the remaining mesenteries. To test this hypothesis, we performed *in vivo* tissue tracking experiments in juveniles overexpressing *Kaede* mRNA. KAEDE is a fluorescent protein that can be permanently photoconverted from green to red by exposing the expressing cells of interest to UV light [38]. For our tissue tracking experiments, we photoconverted three distinct regions of interest at 24 hpa: (1) the epithelia central to the amputation site (Figure 8a); (2) the epithelia lateral to the amputation site (Figure 8b); or (3) the oral tip of the mesenteries (Figure 8c).

It is important to note that when we convert the oral tip of the mesenteries, the laser must pass through the lateral epithelia and this region is also converted. After conversion we analyzed the location of the converted red fluorescence at eight days post-amputation (dpa) using spectral confocal imaging (see materials and methods). Spectral imaging measures the complete fluorescent spectrum of each pixel and matches these to pre-calibrated profiles. In our experiments we calibrated the profiles to detect converted Kaede, unconverted Kaede, and endogenous fluorescence. We found that the central epithelia of the amputation site gave rise to the tentacles in 19 out of 19 cases (Figure 8a,a’). Additionally, this region also gave rise to the mouth (oral-most part of the pharynx) in 15 out of 19 cases (four cases were undetermined) (Figure 8a,a’). The lateral epithelia remained in the lateral tissues after regeneration in four out of 11 cases and in the tentacles in seven out of 11 cases (Figure 8b,b’,b’’). In neither central epithelia nor lateral epithelia conversions did we observe converted cells contributing to the pharynx. In the case of photoconverted mesenteries we observed converted KAEDE-expressing tissues in the newly formed pharynx in seven out of seven cases (Figure 8c,c’; Supplementary Figure S5). These animals also displayed converted tissues in their lateral epithelia and/or tentacles corresponding to the point of the laser entry during the conversion process. Since the lateral tissue remains in the lateral regions or ends up in the tentacles during regeneration but not the pharynx, we conclude that the converted tissue observed in the pharynx is indeed from the oral tip of the mesenteries, confirming our initial hypothesis (Figure 8c,c’; Supplementary Figure S5).

### 2.7. De Novo Transcription Is Induced First in the Gastrodermis at the Amputation Site

**Figure 9:**
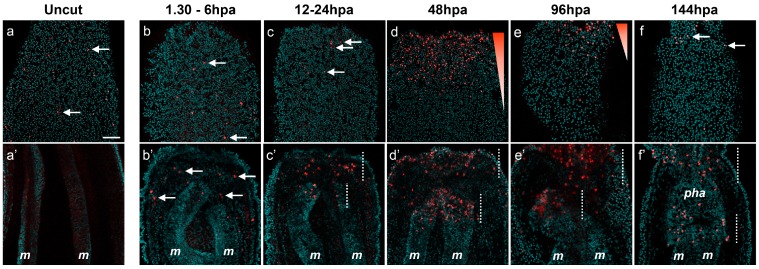
*De novo* transcription at the amputation site of the regenerating polyp. Overlap of confocal images showing *de novo* transcription (EU) in red and nucleus (DNA) staining in cyan in the oral epitheliums (**b**–**f**) and gastric cavity (**b’**–**f’**) of the amputated juvenile polyp. The uncut control is in (**a**–**a’**). The white arrows in **a**,**b**,**b’**,**c**,**f** show the cells that are undergoing *de novo* transcription. The white dotted lines in (**c’**–**f’**) show the regions in the body gastric cavity that are undergoing massive *de novo* transcription, the oral part of the mesenteries, and the epitheliums. All animals are oriented with the amputation site to the top. Scale bar in (**a**) is 20 μm and applies to all Figure 9 and Supplementary Figure S6.

In order to reform missing body parts, an injured organism requires rapid activation of rRNA and tRNA transcription for proper protein biosynthesis of existing or new transcribed mRNA, as well as for cellular proliferation [39,40]. To characterize the transcription in *Nematostella*, we used EU-Click-it chemistry (Life Technologies, Carlsbad, CA, USA) to detect *de novo* transcription in *Nematostella* after amputation (Figure 9).

This technology allows the visualization of EU (Ethynyl Uridine), a modified Uridine analog, incorporated into nascent RNA [41]. In uncut controls, *de novo* transcription is barely detectable and only a few cells were EU-positive (EU+) throughout the body epithelia (Figure 9a,a’). After sub-pharyngeal amputation, between 1.30 and 24 hpa, the *de novo* transcription pattern is similar to controls in regard to the epithelia (Figure 9b,c; Supplementary Figure S6(Aa,Aa’)). However, in the same time frame, some EU+ cells start to emerge in increasing numbers in the internal oral tissues such as the mesenteries and gastrodermis (Figure 9b’,c’). At 48 hpa, a strong EU+ signal is detected in the oral epithelia, both the ectodermis and gastrodermis, and the oral-most part of the remaining mesenteries (Figure 9d,d’; Supplementary Figure S6(Ac,Ac’)). At 96 hpa, EU+ cells are present in the elongating tentacles with the exception of the tentacle tips (Supplementary Figure S6Ba). Staining progressively decreases in the oral epidermis but remains dense in the oral gastrodermis and in the oral-most part of the mesenteries where the new pharynx is developing (Figure 9e,e’). In the fully formed pharynx, at 144 hpa, only a few EU+ cells remain at the base of the tentacles and in the lower part of the pharynx (Figure 9f,f’; Supplementary Figure S6(Ae,Ae’)).

### 2.8. Inhibition of Transcription or Proliferation has Different Effects on Regeneration

In order to determine the role of *de novo* transcription during regeneration in *Nematostella*, we treated amputated juveniles with the antibiotic Actinomycin D (AMD), an inhibitor of DNA-primed RNA synthesis [39]. In untreated controls, EU+ cells are massively detected at 48 hpa. As expected, at the same time-point no staining was observed in the regenerating juveniles that were treated with AMD from 36 to 48 hpa (Supplementary Figure S7a,b). Interestingly, we also observed that cell proliferation and regeneration were inhibited in AMD-treated animals (Supplementary Figure S7c,d).

**Figure 10:**
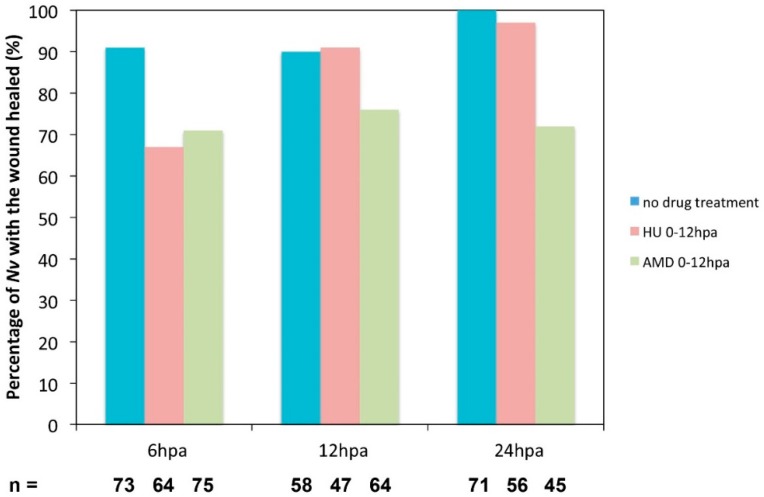
Effect of the inhibition of cell proliferation or transcription on the wound healing process. Hydroxyurea (HU; orange) was used at 20 mM to block cell proliferation, and Actinomycin D (AMD; green) was used at 10 ug/mL to block transcription. Both drugs were used in a time window from 0 to 12 hpa. The compression assay was performed at 6, 12, or 24 hpa.

**Figure 11:**
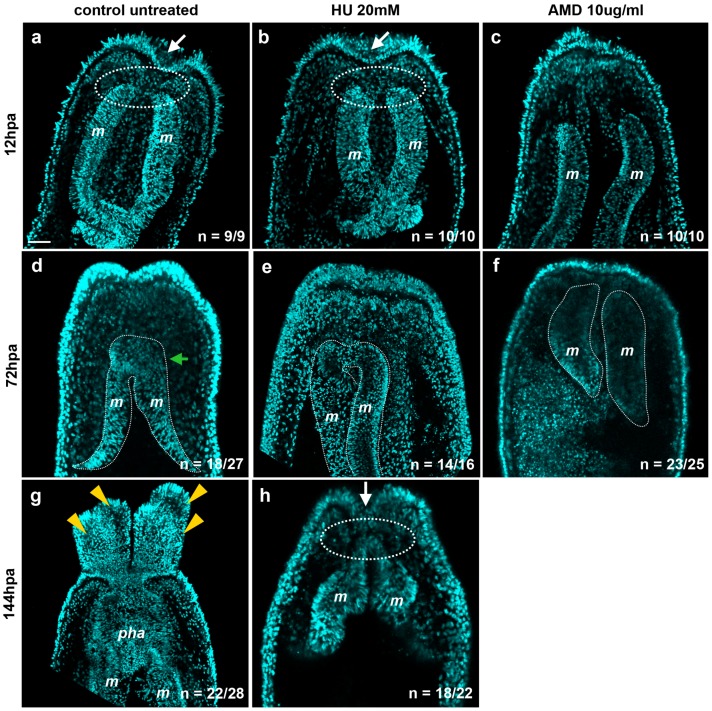
Inhibition of *de novo* transcription blocks regeneration at an earlier step than inhibition of cell proliferation. The experimental amputated polyps were treated with hydroxyurea (HU) to block cell proliferation or with Actinomycin D (AMD) to block *de novo* transcription from 0 to 144 hpa and analyzed at the indicated time-points. Confocal images showing the morphological phenotype using nuclear (DNA, cyan) staining in regenerating control (**a**,**d**,**g**) or experimental (**b**,**c**,**e**,**f**,**h**) polyps at 12 hpa (**a**–**c**); 72 hpa (**d**–**f**); or 144 hpa (**g**,**h**); The white arrows in (**a**,**b**,**h**) show the characteristic depression of the epithelium at the amputated site that correlates with the initiation of the contact between the remaining mesenteries and the surrounding epithelia (circle white dotted line in (**a**,**b**,**h**)); This depression and contact initiation are absent in the polyps treated with AMD (**c**). The green arrow in (**d**) indicates the forming pharyngeal lip. The white dotted lines in (**d**) to (**f**) indicate the mesenteries. The yellow arrowheads in (**g**) indicate the tentacles. *m*, mesenteries; *pha*, pharynx. All animals are oriented with the amputation site to the top. Number of cases for the representative phenotype are in white at the bottom right of each image. Scale bar in (**a**) is 20 μm and applies to all Figure 11.

A recent study has used hydroxyurea (HU) to efficiently block proliferation and regeneration in *Nematostella* [7]. However, nothing is known about the precise phenotype caused by the inhibition of cellular proliferation during regeneration. We thus amputated juveniles below the pharynx, treated them with either AMD or HU from 0 to 144 hpa, and scored wound healing success in addition to the exact stage at which regeneration was blocked using the above-described assays and morphological landmarks at 12, 72, and 144 hpa (Figure 10; Figure 11).

We used the compression assay we described above (Figure 4) to assess wound healing under these experimental conditions. At 6 hpa, wound healing is delayed in a fraction of the HU-treated juveniles (33%, *n* = 58). At 12 hpa, almost all of the animals treated with HU are completely healed (91%, *n* = 47), although the process was delayed by approximately 6 h (Figure 10). However, in AMD-treated juveniles, 29% (*n *= 71) are not healed at 6 hpa, and a similar fraction remains open at 12 hpa (24%, *n* = 56) as well as 24 hpa (28%, *n* = 45) (Figure 10). This observation suggests that wound healing is not blocked but delayed when cell proliferation only is inhibited. However, when both *de novo* transcription and cell proliferation are blocked with the AMD treatment, wound healing does not occur in a subset of animals.

Interestingly, we observed two strikingly different phenotypes at 12 hpa in the HU- *versus* AMD-treated juveniles. Similar to controls at 12 hpa, HU-treated polyps progress to Step 1 when the mesenteries are fused together and enter in contact with the surrounding epithelia at the amputation site (Figure 11a,b). In addition, a characteristic depression is present in the epithelia at the amputation site in the control as well as in HU-treated polyps (Figure 11a,b). However, the AMD-treated regenerating juveniles appear to have been blocked in Step 0, right after the amputation, when the mesenteries are neither in contact with one another, nor with the epithelia of the amputation site (Figure 11c). The characteristic depression in the epithelia at the amputation site is absent as well.

To further characterize the phenotypes resulting from AMD or HU treatment, we assessed pharynx formation using the appearance of 488+ as a proxy at 72 hpa. In untreated regenerating juveniles, the 488+ fluorescence is observed in 62% (38 out of 61 cases). In AMD-treated juveniles, we never observed 488+ in 100% (52 out of 52 cases). We obtained similar results in HU-treated polyps in which 488+ never becomes detectable in 85% (53 out of 62 cases). In addition, no pharyngeal lip or tentacle bulbs were visible in either of the treatments (Figure 11e,f) as the polyps resulting from AMD or HU treatment remain blocked at Step 0 or Step 1, respectively. These data show that for both the inhibition of *de novo* transcription or cell proliferation, the pharynx never starts to form, suggesting that cell proliferation is required for pharynx formation.

At 144 hpa, HU-treated polyps still remain blocked at Step 1 (Figure 11h) compared to the controls in which a fully formed pharynx is present (Figure 11g). At 144 hpa, AMD-treated polyps were highly opaque and degraded (data not shown), suggesting a lethal effect of long-term inhibition of transcription. All together these results show that cell proliferation is required for both pharynx formation and tentacle elongation.

## 3. Discussion

### 3.1. Morphological and Cellular Landmarks for Oral Regeneration in Juveniles

**Figure 12:**
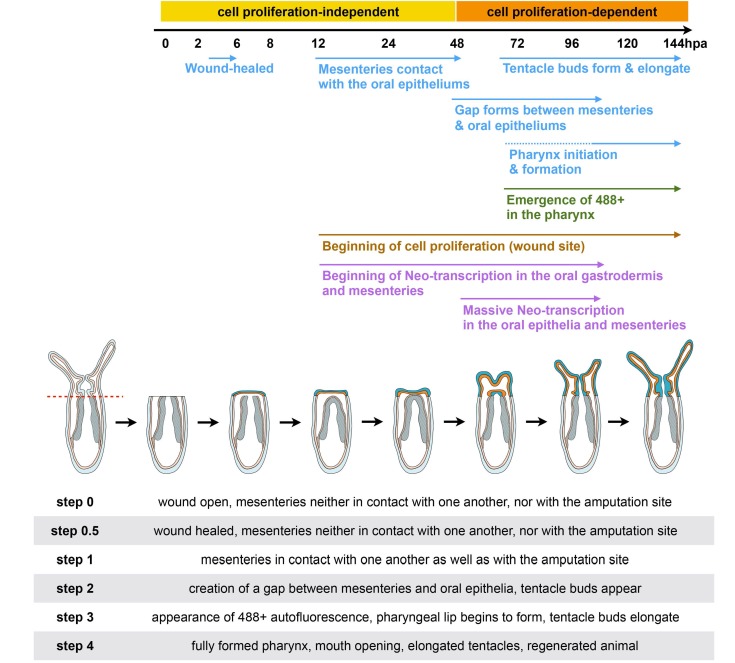
Diagram summarizing the morphological and cellular events underlying oral regeneration in the sea anemone, *Nematostella vectensis.* The table below the illustration provides definitions for the various regeneration steps.

In the present study, we describe in detail the morphological and cellular landmarks for regeneration (Figure 12). We also present *in vivo* tools that can be used to assess wound healing and pharynx formation in juveniles after sub-pharyngeal amputation.

From a morphological point of view, we observe a stereotypic timeline of events: wound healing is completed after 4–6 hpa. Then, the mesenteries fuse together and contact the amputation site at about 12–24 hpa. This is followed by the emergence of a gap between the mesenteries and the wound site between 60 and 72 hpa. This space allows proper pharynx formation around 72–96 hpa by the means of rearrangement of pre-existing mesenteries and cell proliferation. This characteristic behavior of the mesenteries to form the pharynx is accompanied by tentacle bud formation and elongation to form a juvenile that is indistinguishable from its uncut siblings. Along with the morphological timeline, we further describe *de novo* transcription first in the gastrodermis and mesenteries at 12 hpa at the amputation site. Then, a massive wave of *de novo* transcription is activated at 48 hpa in the epidermis, gastrodermis, and in the mesenteries at the amputation site. Similarly, cellular proliferation is first detected at 12 hpa at the wound site and increases progressively over time. It is detected in the oral parts of the mesenteries at 24–48 hpa and remains at high levels until regeneration is achieved.

While these morphological landmarks can be used to define steps of regeneration, none of them can be associated with precise temporal information. This is because within a given batch of animals we observed that regeneration speed varies considerably and not all animals reach a given intermediate step (e.g., pharynx formation) at the same time (see table at the bottom of the Figure 6). However, the highest variation in timing seems to occur around 60–72 hpa. At earlier time points, the batch of regenerating juveniles seems more homogenous in their regeneration stages.

Our present study describes in detail the morphological and cellular events that are initiated either early (0–24 hpa) or late (60–96 hpa) during regeneration. In fact, we did not observe specific morphological or cellular events that are initiated in the period spanning this gap. However, it is known that cellular proliferation progressively increases [7] during this time frame. This suggests important cellular activity and possibly specification events that will subsequently give rise to the new body structures. One way to further characterize the regenerative timeline would be to use gene reporters. *Nematostella* is amenable for gene editing [42] and thus, to further develop this system for regenerative studies, an effort needs to be made in the development of transgenic lines that express genes during a given time span. These could not only highlight the beginning or the end of wound healing or the reformation of a given structure, but perhaps also indicate events that are currently not yet definable using morphology only (e.g., between 24 and 60 hpa). Initial efforts have been made to determine the genes involved in wound healing [34] that could be used for such approaches and this work should be expanded to other regenerative phases.

### 3.2. In Vivo Assays to Assess Wound Healing Success and Reformation of the Pharynx

We developed an *in vivo* assay to determine the success of wound healing after bisection of juveniles by taking advantage of the existence of nematosomes, free circulating aggregations of cells/cnidocytes. Upon compression of the animal with tweezers, the nematosomes exit the body cavity at the amputation site during the process of wound healing and exit through the aboral pore once wound healing is completed. However, we currently cannot exclude the fact that a reformed extracellular matrix or massive production of mucus may prevent the nematosomes from leaking through the wound site during the compression assay before the wound tissue is actually closed.

Unfortunately, the same assay cannot be used to determine the exact timing of mouth re-opening as in uncut juveniles the nematosomes are released through the aboral pore in all cases (100%, *n* = 41). This observation suggests that the fully developed mouth contracts strongly or that the connection between the body cavity and the tentacles disperses and decreases the mechanical forces that the forceps apply to the mouth region. We also tried to assess the timing of mouth opening by feeding the juveniles. However, the size of the animals (100–150 μm) requires that we feed them with smashed brine shrimp, which makes ingestion very heterogeneous within a batch and very difficult to assess. Therefore, detailed morphological and cellular analyses are required to determine the timing of mouth reopening in juveniles (Figure 6e).

In addition, we observe that an endogenous green fluorescence (488+) that is potentially associated with nascent nematosomes is detected ubiquitously in freshly fed animals but localized to the pharynx in juveniles that were starved for one or two weeks, reflecting potentially different metabolic states. We show that hand-sorting uncut animals by size (and health) or bio-sorting them by size and 488+ intensity/localization results in a similar proportion of 488+ and 488− at 72 and 120 hpa (Table 2). We conclude that the Biosorter system is a good way to select a homogenous batch of *Nematostella* polyps with high confidence from a batch of thousands of juveniles. These can be used for experimentation as has been done in other invertebrate and vertebrate models such as *C. elegans* or *D. rerio* [43,44]. In addition, we show that this 488+ correlates with the initiation of pharynx formation during regeneration (Figure 7(Ae–af’)). As a consequence, we successfully used this endogenous fluorescent landmark to assess *in vivo* the reformation of the pharynx in regenerating juveniles after sub-pharyngeal amputation.

Both of the assays we present in this study, compression and emergence of 488+, enable us to determine precise events without having to fix and stain the animals. Thus, the same batch of animals can be followed over the course of the regeneration process. The perturbation experiments we carried out (AMD and HU), and for which we used the *in vivo* assays to determine the timing of the wound healing and the regenerative progression, further underline that these approaches are very useful in juveniles. Furthermore, adults also contain a large amount of free circulating nematosomes, display a strong and very characteristic red endogenous fluorescence in the pharynx caused by the expression of NvFP-7R (not detected in the four-tentacle juveniles), and exhibit green endogenous fluorescence throughout the body and the tentacles [45]. Thus, it would be interesting to use the nematosomes as well as the re-emergence of the pharyngeal NvFP-7R as unbiased assays to determine wound healing and pharynx formation during adult oral regeneration.

### 3.3. Sequence and Origin of the Regenerating Body Parts

We show that juvenile and adult *Nematostella* do not display any differences in the timing of regeneration or the requirement of cellular proliferation after sub-pharyngeal amputation. Thus, it would be crucial to extend this analysis and perform a systematic comparison of the isolated body parts in juveniles and adults. This could identify potential age-related differences or a complete conservation of the cellular (and potentially molecular) mechanisms underlying whole-body regeneration in *Nematostella* juveniles and adults. This is of particular importance since in *Hydra*, the mechanism involved in head regeneration differs depending on the region of amputation (sub-tentacle crown *vs.* mid-body) [9].

We chose to analyze regeneration in juveniles for the ease of imaging morphological details that are more difficult using adult tissues (due to their opacity, size, and fluorescence). However, the morphological characteristics and the staging system we describe for juvenile sub-pharyngeal regeneration cannot be simply extrapolated to adult regeneration and, in particular, it is not intended to replace or extend the existing staging system for regeneration from isolated adult physa in *Nematostella* [30].

In later study, Bossert and colleagues propose (based on macro photographs) that the regenerating adult mesenteries emanate from the most aboral region of the newly formed pharynx during oral regeneration from the isolated adult physa [30]. In contrast, using detailed confocal microscope data and *in vivo* fate-mapping experiments, our present study shows that, in the juveniles regenerating from sub-pharyngeal amputation, the most oral tissues of the mesenteries contribute to re-form a new pharynx.

The precise mechanisms by which mesenteries are formed during embryonic and larval development are poorly described. A first report proposes that these structures are formed by a combination of the pharyngeal ectoderm and body wall endoderm (Aman and Technau, unpublished in [46]). These observations suggest that in the regenerating polyp resulting from adult physa isolation, the mesenteries form in a similar way to those that form during embryonic development, from the already formed pharynx (the origin of which is unclear) [30]. In the regenerating juvenile resulting from sub-pharyngeal amputation, the already present mesenteries are able to give rise to the lost pharynx (data observed in our present study). In order to gain greater insight into the plasticity of pharyngeal and mesenterial fates in all three contexts (embryogenesis, regeneration from isolated physa, or after sub-pharyngeal amputation), further studies focusing on the tissue/cellular molecular mechanisms underlying the origins of these tissues are crucial.

### 3.4. Importance of De Novo Transcription and Cell Proliferation during Regeneration

A previous study on the wound healing process in *Nematostella* showed that mRNA transcription for specific genes is upregulated as early as 1 h post-injury and that the wound signal seems to initiate from the gastrodermis [34]. Interestingly, we also observed a few dispersed EU+ cells, indicative of *de novo* transcription, in the gastrodermal tissues at the amputation site as early as 1.30–6 hpa. It is important to note that even if the size of the injury between these two studies ([34] *vs.* our present study) is different, the beginning of *de novo* transcription seems to correlate.

In the last part of our study, we used the described morphological landmarks as a regeneration staging system, in addition to our *in vivo* assays, to assess in detail the phenotypes obtained under experimental conditions. We showed that inhibition of *de novo* transcription or cell proliferation between 0 and 12 hpa delays or blocks wound healing, but only in the minority of cases. These data are consistent with the timing of *de novo* transcription and cell proliferation, which begin noticeably at 12 hpa, followed by massive waves at the amputation site between 24 and 48 hpa. However, we also showed that the phenotypes observed at 12, 72, and 144 hpa after AMD or HU treatments in regenerating juveniles are strikingly different. The HU treatment shows that the wound healing and early (Steps 0–1) of *Nematostella* regeneration are independent of cell proliferation. Contrary to this, the AMD treatment shows that the *de novo* transcription is required for those same early steps: fusion of the mesenteries to one another and to contact the epithelia at the amputation site (Steps 0–1). These treatments, HU or AMD, block all subsequent regeneration (Steps 2–4) as indicated by the absence of 488+ re-appearance, pharynx formation, and tentacle elongation. These observations strongly suggest that *de novo* transcription is required for the initial tissue dynamics of regeneration (from Steps 0–1), and that cell proliferation is required for later steps (Step 2–4) such as pharynx formation and tentacle elongation.

## 4. Materials and Methods

### 4.1. Animal Culture

*Nematostella vectensis* were cultured at the Institute for Research on Cancer and Aging, Nice (IRCAN) of the University of Nice. Adult and juvenile animals were cultured in 1/3X ASW (Artificial Sea Water; Tropic-Marin Bio-actif system sea salt (Tropic-Marin, Wartenberg, Germany) and maintained at 17 or 22 °C for adults and juveniles, respectively. Adults were fed five times a week with freshly hatched artemia. To obtain juveniles, spawning was carried out as described in [21]. Juveniles for cutting experiments were used six weeks after fertilization. Two-week-old juveniles were fed once a week for two weeks with 1 mL of smashed artemia and then starved for two weeks in order to minimize contamination/background caused by food particles.

### 4.2. Animal Bisection

Juveniles or adults were relaxed by adding 1 mL of 7.14% MgCl_2_ in 5 mL 1/3 ASW and placed on a light table for 10 to 15 min. Six-week-old polyps were cut using a microsurgery scalpel n°15 (Swann-Morton, Sheffield, UK). Each cut was performed perpendicular to the oral–aboral axis of the body column.

### 4.3. Compression Assay

A circle was drawn on a slide with a hydrophobic pen (Dako Pen, Dako) and the relaxed regenerating polyps were placed on this circle in a droplet of 1/3 ASW + MgCl_2_ 7.14%. The polyps were then compressed laterally with the tweezers 3C GRIP (Outils Rubis SA, Stabio, Switzerland). and the expulsion of the nematosomes was assayed. The wound was considered closed when, under pressure, the nematosomes did not leak through the amputated oral part. In this case, the nematosomes either leaked through the aboral opening or did not leak at all. The wound was considered as non-closed when nematosomes were expelled through the oral region. The compression rate is difficult to measure because of the variability among the polyps of the same batch (diverse sizes, metabolic rates, robustness of their tissues). However, to standardize the process, the compression was maintained and accentuated until the nematosomes leaked. In some cases, compression was increased to its maximum and the two tips of the tweezers met.

### 4.4. Staining

After relaxing adults or juvenile polyps in MgCl_2_ for 10–15 min, animals were fixed in 4% paraformaldehyde (Electron Microscopy Sciences #15714, Hatfield, PA, USA) in 1/3 ASW for 1 h at 22 °C or overnight at 4 °C. Fixed animals were washed three times in PBT 0.2% (PBS1X + Triton 0.2%). To analyze the general morphology, Hoechst staining (Invitrogen #33342, Carlsbad, CA, USA) at 1/5000 was used to label the DNA/nucleus, and BODIPY^®^ FL PhallAcidin 488 (Molecular Probes #B607, Eugene, OR, USA) staining was used at 1/200 to label actin microfilaments (cell membranes and muscle fibers).

### 4.5. Cell Proliferation (EdU) and De Novo Transcription (EU) Detection

To detect cellular proliferation Click-it EdU (5-ethynyl-2′-deoxyuridine) kits (Invitrogen #C10337 or #C10339, Carlsbad, CA, USA) were used following the protocol from [7]. The EdU was used at 300 uM following the protocol from [7]. To detect *de novo* transcription Click-it EU (5-ethynyl-2′-uridine) kit (Invitrogen #C10337, Carlsbad, CA, USA) was used following the manufacturer's protocol. EU was used at 1 mM.

### 4.6. Bio-Sorting

The Biosorter system (Union Biometrica, Holliston, MA, USA) equipped with the 2000 μm FOCA flow cell was calibrated with 500 μm beads. Using the Flowpilot software (Union Biometrica), polyps were analyzed for their morphology using the extinction coefficient, their length was analyzed using the Time Of Flight (TOF) between two sensors in the FOCA, and their green endogenous fluorescence intensity (488+) was emitted after a 488-laser excitation. For each animal, 488+ was characterized by measuring the intensity, the width, and the height of the peak in real time on live animals. The debris and small animals were excluded from the analysis based on the TOF and extinction parameters. The 488+ was localized in real time using the Profiler module that scans the profile of the object and integrates the measure of this endogenous fluorescence within this profile, allowing us to determine the relative localization of the 488+ in live polyps. For animal sorting, the optimal drop delay was determined using 500 μm beads and single-animal sorting was performed in 96-well plates filled with 100 μm 1/3 seawater.

### 4.7. Imaging

Live animals were imaged using a protocol described in [16]. The imaging setup was composed of either with a Zeiss Stereo Discovery V8 Discovery or a Zeiss Axio Imager A2 (both Carl Zeiss Microscopy GmbH, Jena, Germany) equipped with a Canon 6D digital camera, triggering two external Canon Speedlite 430 EX II Flashes and controlled by the Canon Digital Photo Professional software (Canon Inc., Tokyo, Japan). Images were edited using Adobe Lightroom 5 and/or Photoshop CS6 software (Adobe Systems Inc., San Jose, CA, USA). Labeled animals were analyzed using a Zeiss LSM Exciter or Zeiss 710 confocal microscope running the ZEN 2009 software (Carl Zeiss Microscopy GmbH, Jena, Germany) from the IRCAN imaging platform (PICMI) or the iBV Platform of Resources in Imaging and Scientific Microscopy (PRISM), respectively. Each final image was reconstituted from a stack of confocal images using Z-projection (maximum intensity or standard deviation) of the ImageJ software (Rasband, W.S., ImageJ, U.S. National Institutes of Health, Bethesda, MD, USA).

### 4.8. Photoconversion Experiment

Fertilized *Nematostella* juveniles were injected with mRNA encoding the photoconvertable Kaede (green to red fluorescence) protein. At six weeks the juveniles were subject to sub-pharyngeal amputation. Then 24 h later, juveniles were relaxed for 10 min with 7.14% MgCl_2_ in 1/3 ASW and mounted between slide and coverslip. The region of interest of the juvenile was photoconverted using a 405 nm wavelength laser on a Zeiss 510 confocal microscope. The specific region was photoconverted with the 405 nm laser at 100% power for 10 iterations (scans) and up to three repetitions at a scan speed less than 10 μs/pixel. When photoconverted, the juveniles were left to recover and regenerate for eight to nine days, then relaxed with 7.14% MgCl_2_ in 1/3 ASW, mounted between slide and coverslip, and analyzed for the red patch of converted Kaede on a Zeiss 710 laser-scanning microscope. To separate photoconverted cells from endogenous fluorescence, using a lambda stack, spectral profiles of converted Kaede-expressing cells, unconverted Kaede-expressing cells, and endogenous red fluorescence were analyzed using a positive and negative control (converted Kaede- and unconverted Kaede-expressing juveniles, respectively). Several different red endogenous fluorescent cells were analyzed to account for variation. Finally, animals were imaged using the online fingerprinting mode and the 34-channel QUASAR detector (Zeiss LSM 710; Zeiss, Gottingen, Germany).

### 4.9. Drug Treatments Hydroxyurea (HU)—Actinomycin D (AMD)

Cellular proliferation was inhibited using HU (Sigma-Aldrich #H8627-5G, St. Louis, MO, USA) and transcription was blocked using the AMD (Enzo Life Sciences Inc. #ALX-260-020-M001, Farmingdale, NY, USA). HU was made up fresh at 20 mM in 1/3× ASW before each experiment. A stock solution of AMD prepared in DMSO and kept at −20 °C was diluted in 1/3X ASW to use at a final concentration of 10 μg/mL prior to each experiment. Each HU or AMD treatment was performed in a final volume of 500 μL 1/3× ASW in a 24-well plate using the adequate controls (1/3× ASW or DMSO). Pharmaceutical drugs were changed every 24 h to maintain their activity for the duration of the experiments.

## 5. Conclusions

In summary, our staging system for oral regeneration after sub-pharyngeal amputation, combined with our *in vivo* assays for wound healing and pharynx formation using naturally existing landmarks, is a precise way to assess the phenotypes resulting from experimental manipulation during regeneration in *Nematostella*. This study also begins to define the cellular and molecular mechanisms underlying the intriguing phenomenon of whole-body regeneration, providing a solid basis for further developing *Nematostella* as a new cnidarian regeneration system.

## Supplementary Materials

Supplementary materials can be found at http://www.mdpi.com/1422-0067/16/12/26100/s1.
