# Supplementary material for: Characterization of Morphological and Cellular Events Underlying Oral Regeneration in the Sea Anemone, Nematostella vectensis
Source: Int J Mol Sci. 2015 Dec 1;16(12):28449–71. doi: 10.3390/ijms161226100 (PMC4691047; doi:10.3390/ijms161226100)
Supplement: Supplementary file 1 [file ijms-16-26100-s001.pdf]

Supplementary Materials: Characterization of Morphological and Cellular Events Underlying Oral Regeneration in the Sea Anemone, *Nematostella vectensis*

Aldine R. Amiel, Hereroa T. Johnston, Karine Nedoncelle, Jacob F. Warner, Solène Ferreira and Eric Röttinger

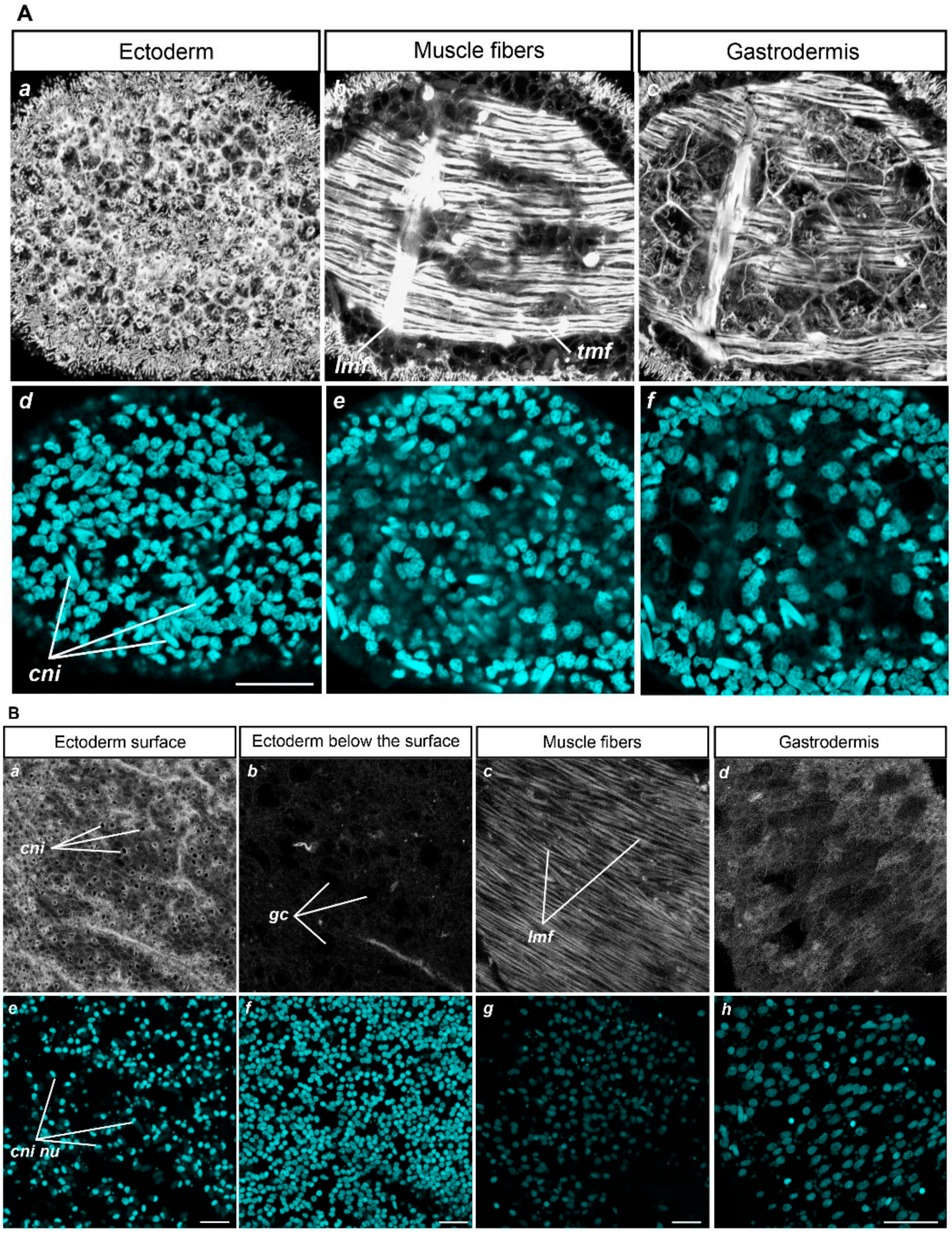

Figure S1. (**A**) Structure of the body wall epithelia in juveniles observed by confocal stacks images. Actin microfilaments (muscle fibers and cell membranes) are stained with PhallAcidin (white, **a**–**c**) and DNA (nucleus) was stained with DAPI (cyan, **d**–**f**). *cni*, cnidocyte(s); *lmf*, longitudinal muscle fibers; *tmf*, transversal muscle fibers. Scale bar in **Ad** is 30 μm; The same scale bar applies for Ae and Af; (**B**) Structure of the body wall epithelia in adults observed by confocal stack images. DNA (nucleus) was stained with DAPI (cyan, **a**–**d**) and actin microfilaments (muscle fibers and cell membranes) are stained with PhallAcidin (white, **e**–**h**). *cni*, cnidocytes; *cni nuc*, cnidocyte nucleus; *gc*, gland cells; *lmf*, longitudinal muscle fibers. Scale bar in (**Be**–**Bh**) is 30 μm.

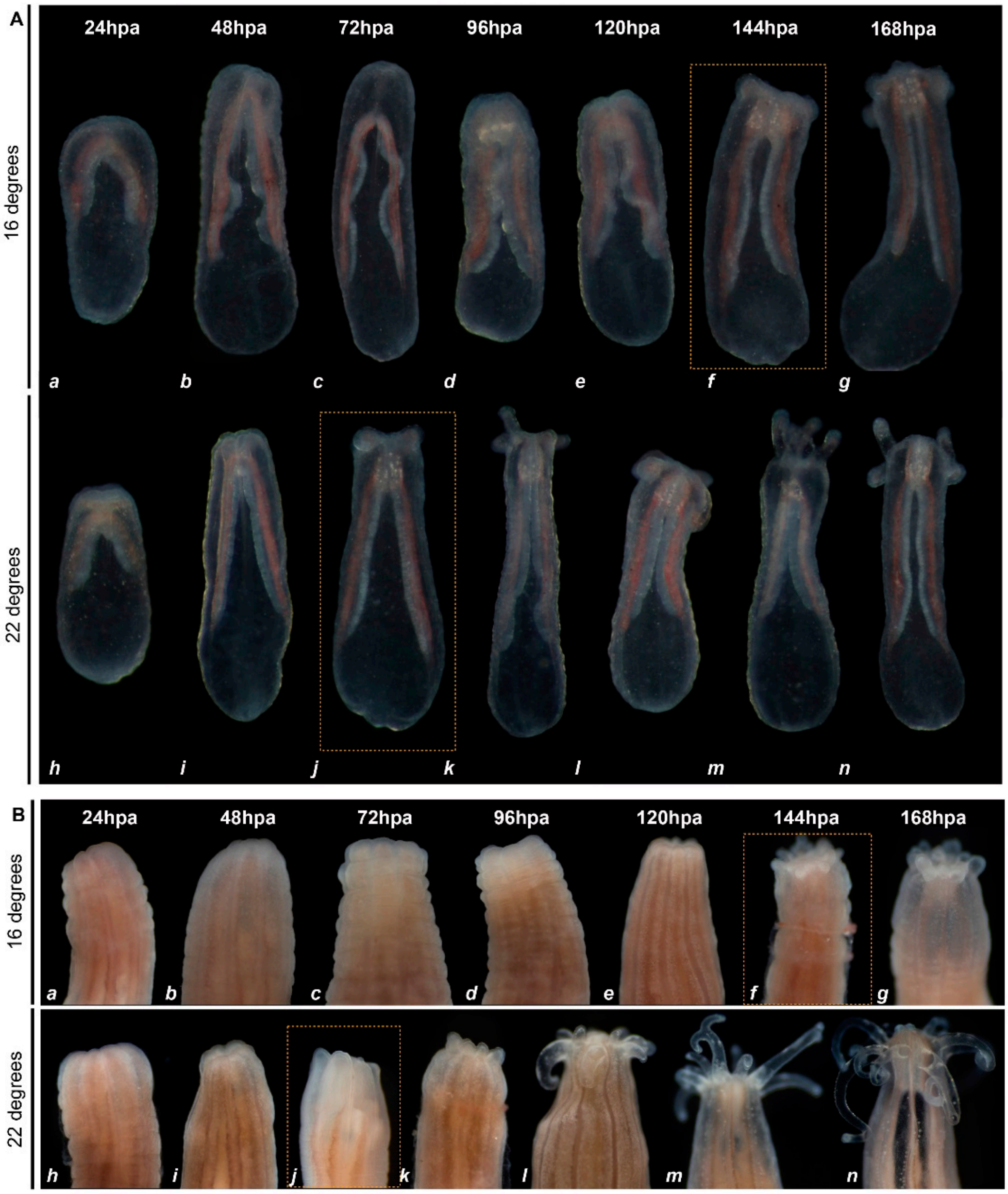

Figure S2. (**A**) Oral regeneration is temperature dependent in juveniles. From left to right: regenerating polyps at 16 °C (**top** panel) and 22 °C (**bottom** panel) at 24 h post amputation (hpa), 48, 72, 96, 120, 144, 168 hpa. The tentacle bulbs and the pharynx are visible starting just 144 hpa at 16 °C and 72 hpa at 22 °C (orange square with dotted line); (**B**) Oral regeneration is temperature dependent in adults. From left to right: regenerating polyps at 16 °C (**top** panel) and 22 °C (**bottom** panel) at 24 h post amputation (hpa), 48, 72, 96, 120, 144, 168 hpa. The tentacles bulbs are visible starting just 144 hpa at 16 °C and 96 hpa at 22 °C (orange square with dotted line).

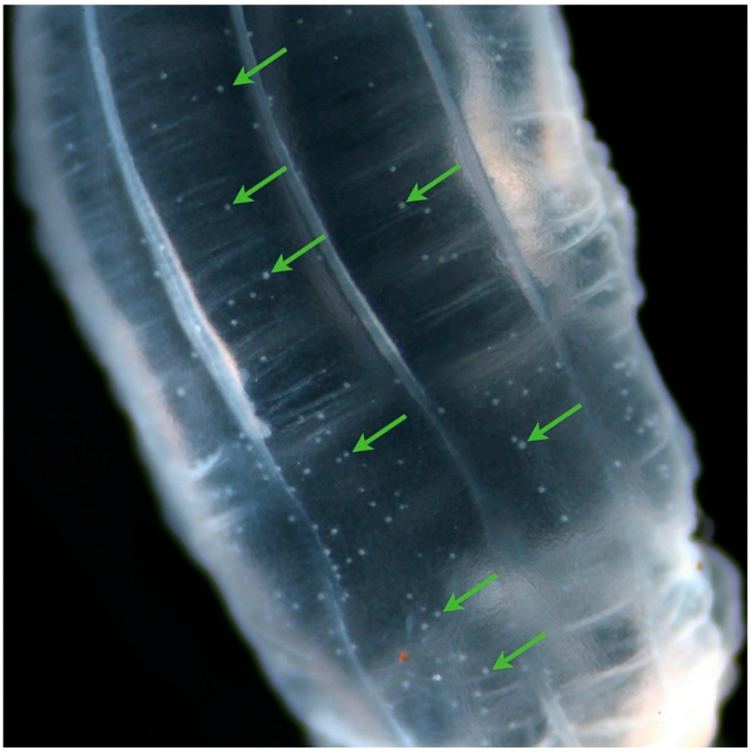

Figure S3. Nematosomes. The white dots indicated by the green arrows are the nematosomes circulating in the *Nematostella* gastric cavity. Nematosomes are used as markers for intragastric fluid dynamics during the compression assay.

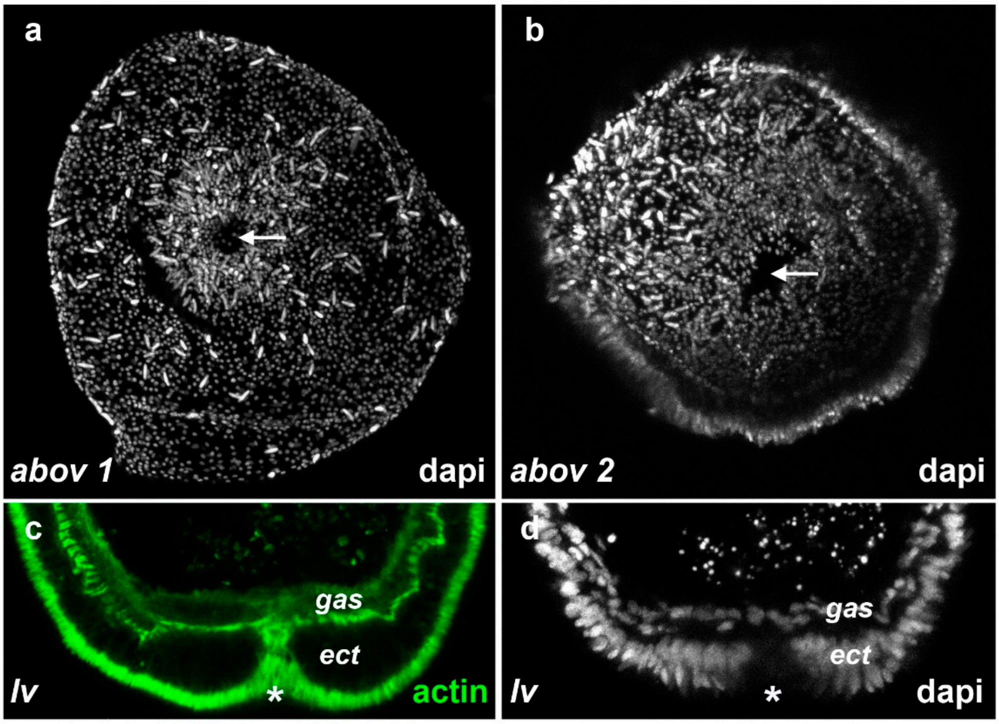

Figure S4. Aboral Pore. Confocal images of the aboral part of the *Nematostella* body on an aboral view (*Abov*) (**a**,**b**) and a lateral view (*lv*) (**c**,**d**); The DNA (nucleus) is labeled with DAPI (white) (**a**,**b**,**d**) and actin (microfilament) is labeled with Phalloidin (green) (**c**); The white arrow (**a**,**b**) and the asterisk (**c**,**d**) show the Aboral pore. *ect*, ectodermal epithelium; *gas*, gastrodermis.

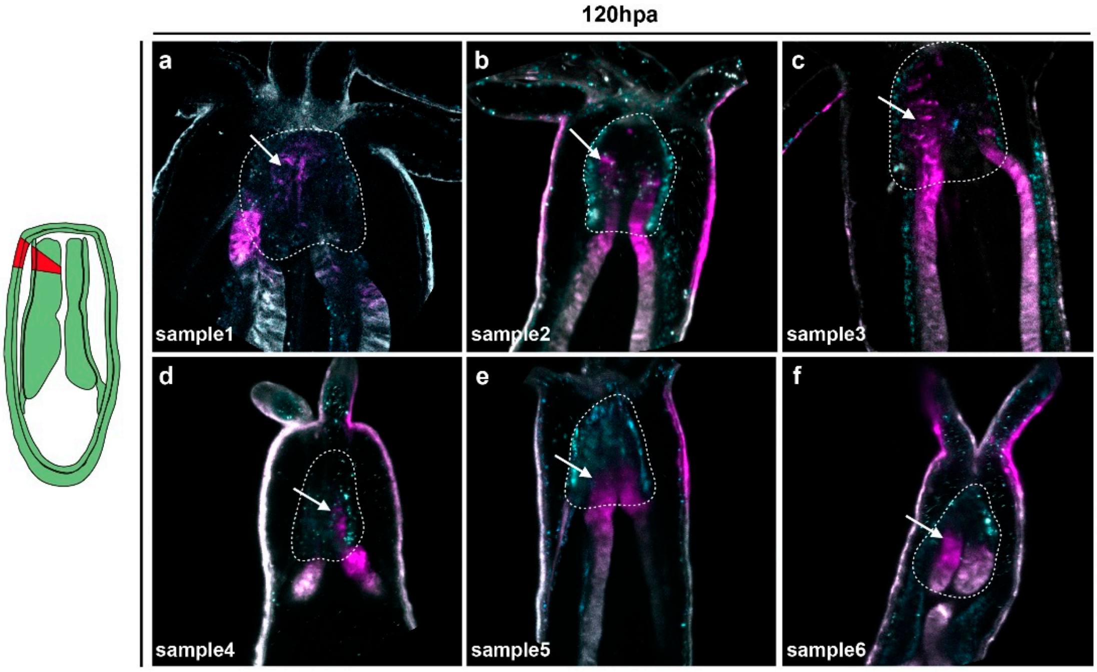

Figure S5. Tissue tracking experiment during regeneration focusing on the seven samples in which the oral most end of the mesenteries were exposed to a UV laser at 24 hpa resulting in permanent photo-conversion of the KAEDE protein in this region of interest. Spectral confocal images of *Nematostella* juveniles expressing Kaede photoconvertible fluorescent protein mRNA at 120 hpa (**a**–**f**). The Keade photo-converted region is represented in magenta and the non-photoconverted region in grey. The endogenous fluorescence is shown in turquoise to help visualize the morphology of the polyp. The photoconversion of the oral tip of the mesentery results in integration of the converted patch into the pharynx (white arrows in (**a**–**f**)). The dotted lines in (**a**–**f**) indicate the position of the pharynx.

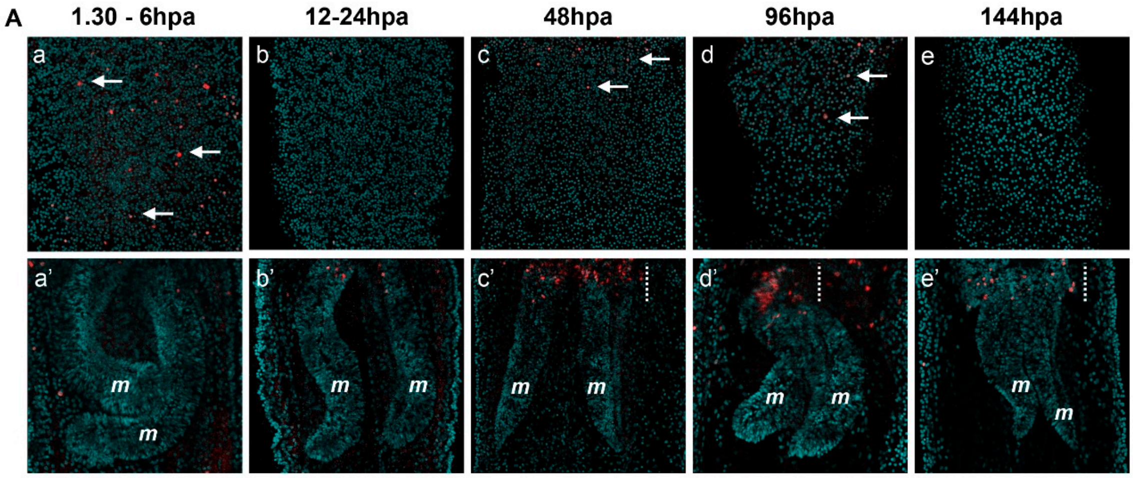

Figure S6. *Cont.*

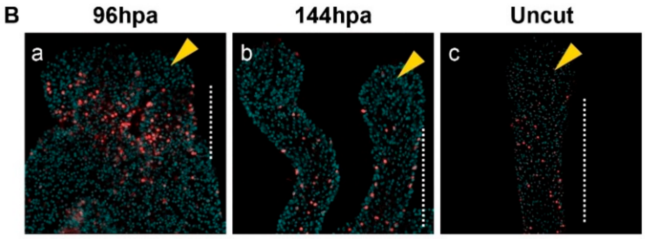

Figure S6. *De novo* transcription in the mid-body (**A**) and tentacle (**B**) region of the regenerating polyp. Overlapping confocal images showing *de novo* transcription (EU) in red and nucleus (DNA) staining in cyan in the mid body epitheliums (**Aa**–**Ae**); gastric cavity (**Aa′**–**Ae′**) and tentacles (**Ba**–**Bc**) of the amputated polyp juvenile; The uncut control is in (**Bc**); The white arrows in (**Aa,Ac,Ad**) show the cells that are undergoing *de novo* transcription; The white dotted lines in (**Ac′**–**Ae′**) show the region in the body gastric cavity that are undergoing massive *de novo* transcription, oral-most part of the mesenteries; The yellow arrowheads in Ba and Bb show the absence of *de novo* transcription in the tips of the regenerating tentacles, similar to the controls in (**Bc**), probably reflecting the high density of differentiated cnidocytes in this specific location. All animals are oriented with the amputation site to the top. *m*, mesentery; *pha*, pharynx.

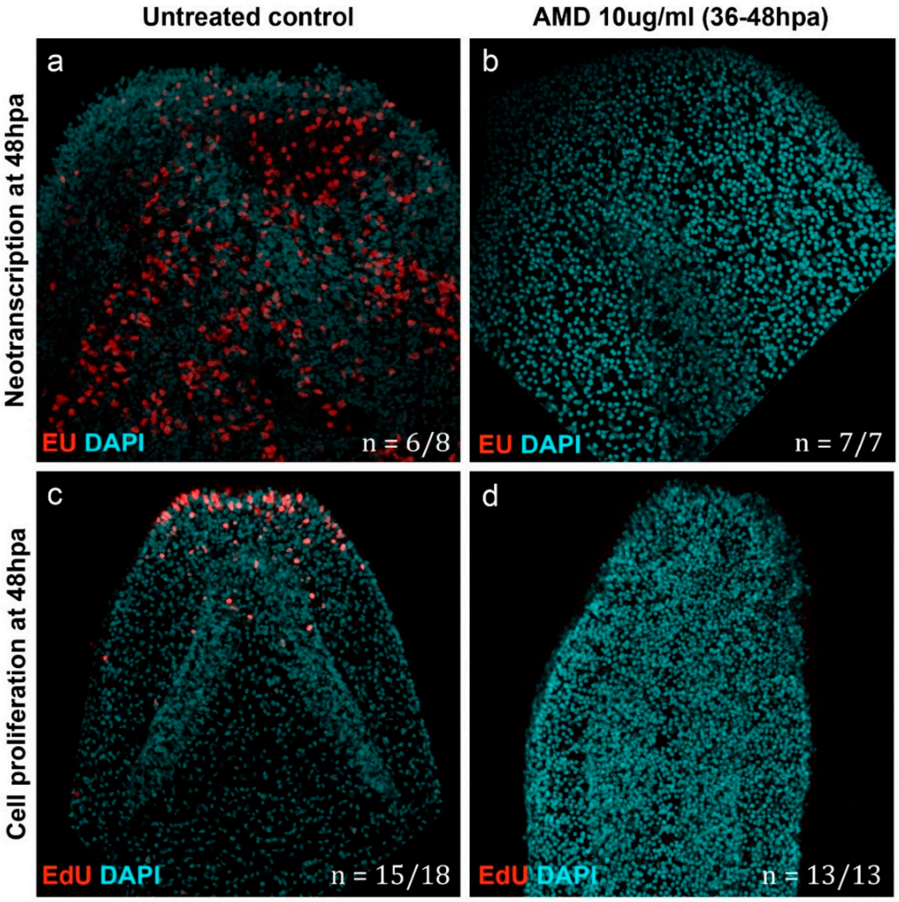

Figure S7. Actinomycine D blocks *de novo* transcription, cell proliferation and regeneration. Overlap of confocal images showing *new transcription* (EU) in red and nucleus (DNA) staining in cyan (**a**,**b**) or proliferating cells (EdU) in red and nucleus (DNA) staining in cyan (**c**,**d**) in the regenerating juvenile at 48 hpa. Controls for *de novo* transcription are in (**a**); and for cell proliferation in (**c**); The experimental juveniles were treated from 36 to 48 hpa with the Actinomycin D (AMD) and *de novo* transcription (**b**) or cell proliferation (**c**) were analyzed. All animals are oriented with the amputation site to the top. Number of cases for the representative phenotype are in white at the bottom right of each image.
